# Recognizing the Common Origins of Dystonia and the Development of Human Movement: A Manifesto of Unmet Needs in Isolated Childhood Dystonias

**DOI:** 10.3389/fneur.2016.00226

**Published:** 2016-12-19

**Authors:** Jean-Pierre Lin, Nardo Nardocci

**Affiliations:** ^1^Guy’s and St Thomas’ NHS Foundation Trust, London, UK; ^2^Fondazione I.R.C.C.S. Istituto Neurologico Carlo Besta, Milano, Italy

**Keywords:** dystonia, developmental dystonia, cocontraction, tonic labyrinthine response, cerebral palsy, Gowers, genetic heterogeneity, phenotypic pleiotropy

## Abstract

Dystonia in childhood may be severely disabling and often unremitting and unrecognized. Considered a rare disorder, dystonic symptoms in childhood are pervasive in many conditions including disorders of developmental delay, cerebral palsy (CP), autism, neurometabolic, neuroinflammatory, and neurogenetic disorders. Collectively, there is a need to recognize the role of early postures and movements which characterize phases of normal fetal, infant, and child development as a backdrop to the many facets of dystonia in early childhood neurological disorders and to be aware of the developmental context of dystonic symptoms. The role of cocontraction is explored throughout infancy, childhood, young adulthood, and in the elderly. Under-recognition of pervasive dystonic disorders of childhood, including within CP is reviewed. Original descriptions of CP by Gowers are reviewed and contemporary physiological demonstrations are used to illustrate support for an interpretation of the tonic labyrinthine response as a manifestation of dystonia. Early recognition and molecular diagnosis of childhood dystonia where possible are desirable for appropriate clinical stratification and future precision medicine and functional neurosurgery where appropriate. A developmental neurobiological perspective could also be useful in exploring new clinical strategies for adult-onset dystonia disorders focusing on environmental and molecular interactions and systems behaviors.

We present a “Manifesto of Unmet Needs in Childhood Isolated Dystonias” (summarized in Panel 1. A Manifesto of Unmet Needs in Isolated Childhood Dystonias in Appendix), partly to encourage recognition of dystonia as an important cause of morbidity in children, to stimulate work capturing how dystonia affects the lives of children by using appropriate outcome measures and to develop new strategies for managing dystonias in a timely fashion.

Dystonia (see Panel 2. Dystonia, Dyskinesia, and Hypertonus in Appendix for current and historical definitions) in childhood may be severely disabling and often unremitting (1). Considered a rare disorder, dystonic symptoms in childhood are pervasive in many conditions including disorders of developmental delay, CP, autism, neurometabolic, neuroinflammatory, and neurogenetic disorders. Collectively, there is a need to recognize the role of early postures and movements, which characterize phases of normal fetal, infant, and child development as a backdrop to the many facets of dystonia in early childhood neurological disorders and to be aware of the developmental context of dystonic symptoms.

The predicament of a child facing a life with dystonia today has improved considerably over the past 20 years. New genetic diagnoses and complex neurosurgical solutions such as deep brain stimulation, highlight as never before, the need for even greater clinical-diagnostic skill to adequately characterize the clinical phenomenology and select the optimal management within an appropriate time-frame. Children with dystonia need a “manifesto” to guide clinical research programs and focus the resources of the clinical and scientific communities.

## There is a Need to Recognize the Importance of Early Experience of Movement for the Fetus and Infant and Environmental Effects on This Early Development

The phenomenon of dystonia can best be understood in the context of the phylogeny (evolution) and ontogeny (development) of the motor system. This approach indicates that dystonic postures and movements leading to twisting and writhing patterns of movement (see Panel 2. Dystonia, Dyskinesia, and Hypertonus in Appendix) are innate features of early human life. A fundamental question may be posed: could the fetus, newborn, or infant develop a motor repertoire if the early default state was one of akinesia?

Nature has already provided ample evidence that the answer is emphatically negative. The akinetic fetus is invariably born with severe contractures, often without appropriately developed joint cavities in what is termed the fetal-akinesia deformation sequence with dire consequences for future survival and motor development (2). Externally acquired mechanisms of paralysis with neuromuscular paralyzing agents (3, 4), effects of maternal tetanus on fetal movements (5), or antibodies to the human fetal neuromuscular junction acetylcholine receptors, produce early akinesia with similar effects (6–8) and genetic defects in neuromuscular junction acetylcholine receptor proteins likewise (9). Immobility may not only impair or abolish joint cavity formation in flaccid paralysis (10) but also profoundly affect bone growth (10), an effect also seen after rigid immobility (11) following brain injuries to the chick embryo. Movement or motion itself is therefore essential for the development of a functional musculoskeletal system in the fetus and infant. In this respect, it is remarkable that the human fetus engages 11 separate patterns of movement of increasing complexity as gestation progresses including “twitches,” “independent limb,” “isolated head” movements or “combination movements,” “quazi-startle” (sudden), or “hand-face” movements as well as “isolated body extension” and “thumb sucking,” long before the onset of breathing movements at 18 weeks gestation (12). These early patterns of movements often following a dermatome–myotome relationship could be seen as fetal precursors of the “geste antagoniste” postures seen in later life, which are so successful in abolishing dystonia.

After delivery, the mammalian motor system is not yet anatomically committed to the adult arrangements. Remarkably, polysynaptic innervation of agonist–antagonist muscle pairs across a limb-joint is altered by the experience of motion (13–17). There then follows postnatal redundant axon elimination in health (14, 15, 17, 18) that occurs abruptly (19). But polysynaptic innervation may persist following external interventions to the muscle–tendon complex, for instance tenotomy in newborn rats, which results in a lack of activity-dependent normal synapse elimination (20). Similar results arise after chronic use of local anesthetics to block nerve conduction, which is associated with persistence of newborn polysynaptic innervation patterns (21, 22). These events may be relevant to ex-preterm or sick neonates who move less while undergoing neuromuscular paralysis for mechanical ventilator support as described above. But it is not known how typical synaptic pruning is influenced by excessive movement.

Increased “reciprocal excitation” in healthy young infants is lost in adults indicating a physiological maturation of the reciprocal stretch reflexes in normal development but this reciprocal excitation is retained in children with cerebral palsy (CP) due to perinatal injuries (23, 24), thus favoring persistence beyond early infancy of cocontraction muscle synergies.

## A Need to Recognize the Link Between Childhood Dystonias and Patterns of Movement and Postures in the Infant and Toddler. This Involves Recognizing the Key Patterns of Emergent Motor Development and Applying Operational Definitions That Enable Recognition of These Key Patterns, Including Understanding the Link Between Developmental Dystonia and Pathological Dystonia at all Ages of Life

In addition to the remodeling of the innervation of agonist–antagonist muscle pairs of the peripheral neuromuscular system, the fetus and infant undergo centrally driven truncal, arm and leg flexor, and extensor postural stages (25).

The cerebral glucose metabolism of the preterm and full-term baby shows that the dominantly flexed postures with apparently “dystonic-athetoid” movements are associated with mainly thalamic and brain stem and cerebellar glucose metabolic activity (26). It can be hypothesized that in developmental terms, persistent flexed trunk and limb postures are dominated by thalamic activity and the term newborn represents “*Dystonic–Athetoid Thalamic Man*.”

By 3–4 months of age, the infant trunk adopts a symmetrically straight posture, there is head control and the back is straight in supported sitting or standing, but the arms and legs are hyperactive in what can only be described as dancing movements, i.e., “physiological or developmental chorea,” representing “*choreiform man”*: parents may even refer to their infant performing the popular Irish dance: “*Riverdance*.”

By 6–8 months, seemingly purposeless chorea is replaced by directed and targeted swatting and kicking movements of arms and legs. This represents the “*Kung-Fu Man*” phase when arms and legs appear to share common skills in accessing targets which occurs before the legs acquire a locomotor role. This pre-locomotor “equipotential” phase of motor abilities in all four limbs underpins the capacity of feet to be used as hands in naturally occurring limb-deficit impairments such as “*phocomelia*” when the arms may be entirely missing and the feet are used for manual tasks. Another description of this phase of motor development could be the “*democratic phase of all four limbs*,” which is only witnessed in the pre-weight-bearing and pre-locomotor phase of human development.

This period of development also coexists with intermittent dystonic posturing including simultaneous leg extension-hip-abduction or adduction postures, equinovarus foot posturing, spontaneous extensor great toes, and fanning of the toes, which are often referred to as “*striatal toes*.”

At 6–10 months, the infant develops the ability to play with the feet and adopt “*ballerina*” poses, often holding the legs straight up in the air and playing with the feet. These postures dissipate soon after the legs are committed to locomotor tasks, either when bottom-shuffling or the development of bi-pedal ambulatory mobility (Lin, unpublished results).

**Figure 1:**
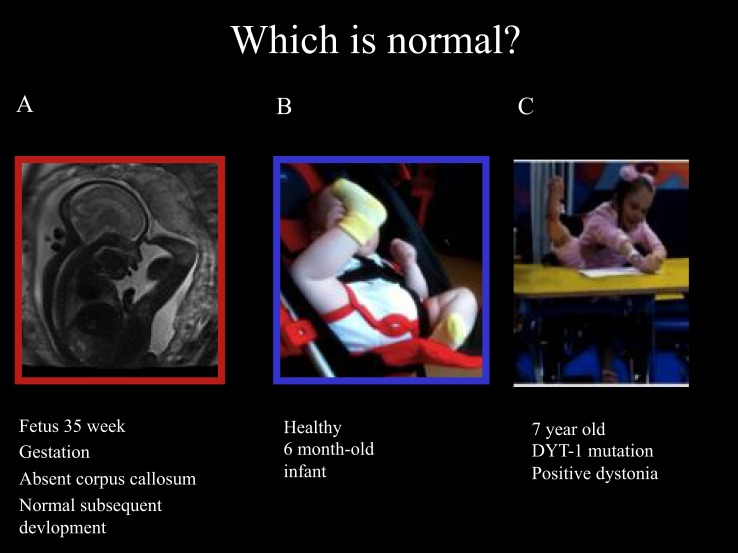
**Fetal, infantile and pathological ballerina posturing dystonia**. Ballerina posture in 35-week gestation fetus with absent corpus callosum and subsequently normal development **(A)**, healthy 6-month old infant **(B)**, and a 7-year old girl with typical-onset isolated monogenic dystonia secondary to the DYT-1 mutation for the past 11 months **(C)**. The dystonia began in the left leg, spread to the right leg enforcing wheel-chair mobility after 6 months then spread to the arms: note shoulders hunched, arms extended to write, and left leg extension under the table with “ballerina” right leg posturing associated with equinovarus posturing at the right ankle. These pictures illustrate that “ballerina posturing” is common in infancy but pathological after the first year of life. The dystonia posturing may be considered a release of formerly dominant movements and postures of the legs before independent floor locomotion, standing, and walking. It is noteworthy that the ballerina posturing of the right leg was abolished as soon as the 7-year old girl regained independent standing 1 month after DBS. This reinforces the link between dystonic postures and functional levels. In this case, a transient regression to infantile posturing is brought about by dystonia.

Ballerina posturing of the legs can also be seen in young children with isolated monogenic dystonia due to the Torsin-A mutation (*DYT*-1). These developmental motor patterns are illustrated in Figure 1, including the fetal leg extension postures and typical infant leg “stereotypies” at 6 months of age and the ballerina posturing in DYT-1 dystonia as an illustration of the link between dystonic postures and movements and early physiological postures and movements.

## An Unmet Need to Recognize the Similarities and Differences Between Developmental Cocontraction, Task-Dependent Cocontraction, the Pathological Cocontraction of Dystonia, Observations of Selective Motor Control, and Surround Inhibition in Children, the Elderly, and in Dystonic Individuals

As briefly reviewed, the embryo, fetus, and young infant must be hyperkinetic to overcome gravity and the risk of early intrauterine and extra uterine deformity, respectively. Another function of hyperkinesia is refinement of the initially clumsy, goal-directed motor behaviors, by harnessing activity-dependent plasticity for the successful transition from “goal-directed movements” (which require conscious direction) to “habitual movements” (27). Habitual movements are internalized, semi-automatic movements that can be executed as part of more complex tasks such as reaching, holding, and using tools, or, for the legs, for ambulatory and propulsive tasks.

In older children and adults, weight-bearing limbs have high inertia and low muscle stiffness whereas limbs for fine, non-repetitive, and complex movements such as our digits have low inertia and high muscle stiffness. This arrangement ensures that our limbs can resist unwanted oscillation using inertia for the legs and intrinsic hand muscle stiffness for the fingers and toes, wrists, and ankles without expending unnecessary energy through muscle activation (28).

### How Does the Motor System Adapt from Infancy to Adulthood?

A reasonable explanatory narrative for these dramatic alterations in motor properties is that in early infancy the muscles and tendons have very high compliance, i.e., reduced stiffness (28), the mechanical–anatomical consequences of which are compensated for by active cocontraction in all tasks, leading to a stiff-jerky, coarse movement repertoire, rather than the skilled-economical motor repertoire of later years. Muscles are also different in infancy and early childhood, reflex muscle twitches being weak and slow to contract and relax (28, 29), contributing to coarse movement patterns on attempting skilled tasks (30). Developmental cocontraction (31), i.e., *developmental dystonia*, compensates for reduced intrinsic stiffness and low limb-inertia in early life (28–30).

Panel 3. Causes of Cocontraction in Children in Appendix summarizes the phases of developmental physiological cocontraction, task-dependent cocontraction, pathological cocontraction, and the return of cocontraction in the infant and the elderly (Figures 4A–D).

### Early Movements and Postures and Cocontraction

#### Developmental Cocontraction and Joint-Synergies in Early Standing and Walking

When an infant first stands, the posture is typically stooped forward, arms abducted, fingers spread, legs widely abducted (spread apart), hips, knees, and ankles flexed, i.e., the “triple flexion” joint-synchrony posture. This triple-flexion muscle activation pattern produces the characteristic “crouch posture” and “crouch gait” of early infancy. However, this crouch posture is also the hallmark of the standing pattern in CP and of course in other later-acquired motor disorders. Because the crouch posture is so tiring (the reader can prove this to themselves by adopting a crouch stance while reading this text), infants and children and young adults may automatically replace ankle dorsiflexion with an *equinus posture*, i.e., by going up on tip-toes, which relieves some of the strain on the knee extensors. Thus, ankle equinus, which mechanically promotes biomechanical knee extension, is less tiring than an ankle crouch (dorsiflexion) posture. But standing in equinus is less mechanically stable than standing plantargrade. Both crouch and equinus gait and stance are associated with muscle cocontraction.

It is well known that infants exhibit stiff and jerky movements when standing and walking with support, which, together with the joint-synchrony posture and movement pattern is underpinned by cocontraction of most trunk and limb muscle groups required for these tasks; gradually developing more fluent and economical movements when supported walking is replaced by unsupported walking, and cocontraction is replaced with a graceful interplay of agonist–antagonist muscles (31).

#### Heel-Strike as a Hallmark of the Mature Gait

The first hallmark of a mature gait pattern is the ‘*heel-strike*’ at initial foot contact. But this seldom occurs before approximately 2 years of life, and then not for all 2-year olds.

The second hallmark of the mature gait is the advent of asynchronous joint patterns during the swing-phase of gait, i.e., *hip flexion-knee-extension-ankle dorsiflexion*, this last also producing the famous heel-strike of a mature gait.

The third hallmark of a mature gait is the contralateral arm swing. In this, the humerus swings forward synchronously with the contralateral femur of the “*swing*” leg. This pattern is similar to the crossed innervation pattern of the quadruped or vertebrate locomotor pattern. This is present when the infant crawls and is suppressed when the infant first stands and takes its first steps, returning with the advent of a mature gait at about 24 months of age.

The arm swing cannot be taken for granted, since it is lost in Parkinsonism when the stooped posture returns, but it is also lost or “buried” in the cocontraction of early bi-pedal mobility.

#### Wrap-Around Muscle Activation Patterns and Motor Development

Sutherland and colleagues (32) found prolonged calf muscle and tibialis anterior-muscle activation during gait in two-thirds of healthy 1-year olds and a third of typically developing healthy 7-year olds. This led to the observation of a “*wrap-around EMG*” pattern of muscle activation throughout the *stance* and *swing* phases of gait (32). This produces an ankle *equinus* pattern, i.e., plantarflexion in terminal swing causing the phenomenon of a tip-toe foot-contact pattern and of toe walking, so common in young infants, and contributing to the tottering, stiff-legged, high-energy, and unstable gait patterns typical of the “toddler” who is notoriously prone to frequent falls.

But as Sutherland discovered, one-third of healthy 7-year olds also exhibited this “wrap-around EMG” pattern of calf muscles leading to a phase of prolonged developmental toe walking (32). This developmental, non-pathological toe walking is conceptually important for our further understanding of pathological dystonia since, as will be demonstrated, it helps our understanding of how the motor system adapts to perturbations and injuries.

#### Developmental Cocontraction with Unfamiliar Tasks in Children and Teenagers

“Fog Posturing” assesses motor immaturity during unfamiliar tasks (33). All 4-year olds and 25% of 16-year olds exhibit involuntary associated postures of face, tongue, arms, or hands during unfamiliar tasks (33): a task-dependent “*overflow*” or dystonia persisting with learning difficulties, motor impairments, or speed of walking. The “Fog Manoeuver,” i.e., asking the subject to walk on their toes, heels, lateral borders, and then the in-step of the feet, also accentuates the presence of latent or task-dependent coactivation patterns often referred to as “overflow.” In the original Fog and Fog (33) report, asking children to open a bullfrog clip also produced associated, involuntary postures. *Of course the transition between a “developmental” overflow and “pathological” overflow is context-dependent, but the inability to suppress overflow at age-appropriate points must be considered part of a pathological spectrum of motor outputs that fall into the category of dystonias*. The Fog manoeuver may be used to accentuate subtle or latent dystonic postures in CP (34) or indeed isolated monogenic dystonias.

### Selective Control (SC) and Surround Inhibition

Lack of selective motor control and surround inhibition in children with CP produces unwanted synergies (35).

Corticomuscular coherence amplitude is inversely related to motor performance in young adults but not modulated in young children and the elderly who have larger, more widely distributed cortical networks (36).

Magneto-encephalography, exploring cortico-oscillatory activity during knee extension tasks in CP children, demonstrated increased mean β*-event-related-desynchronization* (β*-*ERD) during motor planning but weaker γ*-event-related-synchronization* (γ*-ERS*) within primary motor cortex neurons (37).

A lack of selective motor control, excessive plasticity, and loss of surround inhibition is also a hallmark of dystonia (38, 39).

These observations raise important questions regarding the similarities and differences between these apparently different clinical groups.

One interpretation is that the young and elderly have less SC and reduced surround inhibition; a phenomenon shared with CP children and dystonic subjects.

## An Unmet Need to Recognize That Dystonia is Abolished by Sleep in Children and Adults and may Therefore be Used as a Diagnostic Criterion for Dystonia as Well as Therapeutically, for Instance in Status Dystonicus (SD)

One of the first questions a clinician should ask when faced with a child with a dystonic motor disorder should be:

1. “How are your child’s movements and postures affected by sleep?”
2. “How much sleep is your child getting at night?”

The same questions can be raised when other colleagues refer children with motor disorders for further investigation and management.

The exploitation of sleep as a means of “switching off dystonia” is also an important therapeutic concept to be exploited in severe or life-threatening dystonias.

### Dystonia and the Influence of Sleep

Dystonia is usually abolished by sleep and exacerbated by emotion, pain, mental concentration, or intention to move, i.e., by non-specific afferent inputs. Dystonia, Parkinsonian rigidity, tics, chorea, and athetosis are always abolished by sleep (40, 41). The exploration of the influence of sleep should therefore be a focus of all dystonia management plans (42–44) and may be considered the “*poor man’s examination under anesthesia*.”

### SD and Sleep

A crucial use of sleep to “switch off dystonia” in SD can be a very valuable clinical strategy for managing this life-threatening clinical catastrophe, which most commonly occurs in adolescent males with secondary dystonia, often of CP origin (42, 43, 45). The disadvantage of conventional muscle relaxants is that they also provoke respiratory depression and muscle weakness leading to central hypoventilation, shallow breathing, or frank apnoea (42, 43), whereas the hypnotic approach, e.g., with clonidine, can switch off SD by inducing sleep while preserving muscle function and breathing (43).

## The Need to Recognize Dystonia That Pervades CP and Reverse the Over-Investment in the “Spastic Model” of CP in Medical Teaching and Training on Motor Disorders in Childhood Arising from Historical Roots at a Time When Dystonia was Not Commonly Recognized Leading to an Under-Recognition of the True Prevalence of Dystonic CP Including a Reexamination of the Nature of the Tonic Labyrinthine Response (TLR)

### Gowers’ Description of Primary Spastic Paraplegia and the TLR and an Essentially Dystonic Phenomenology

**Figure 2:**
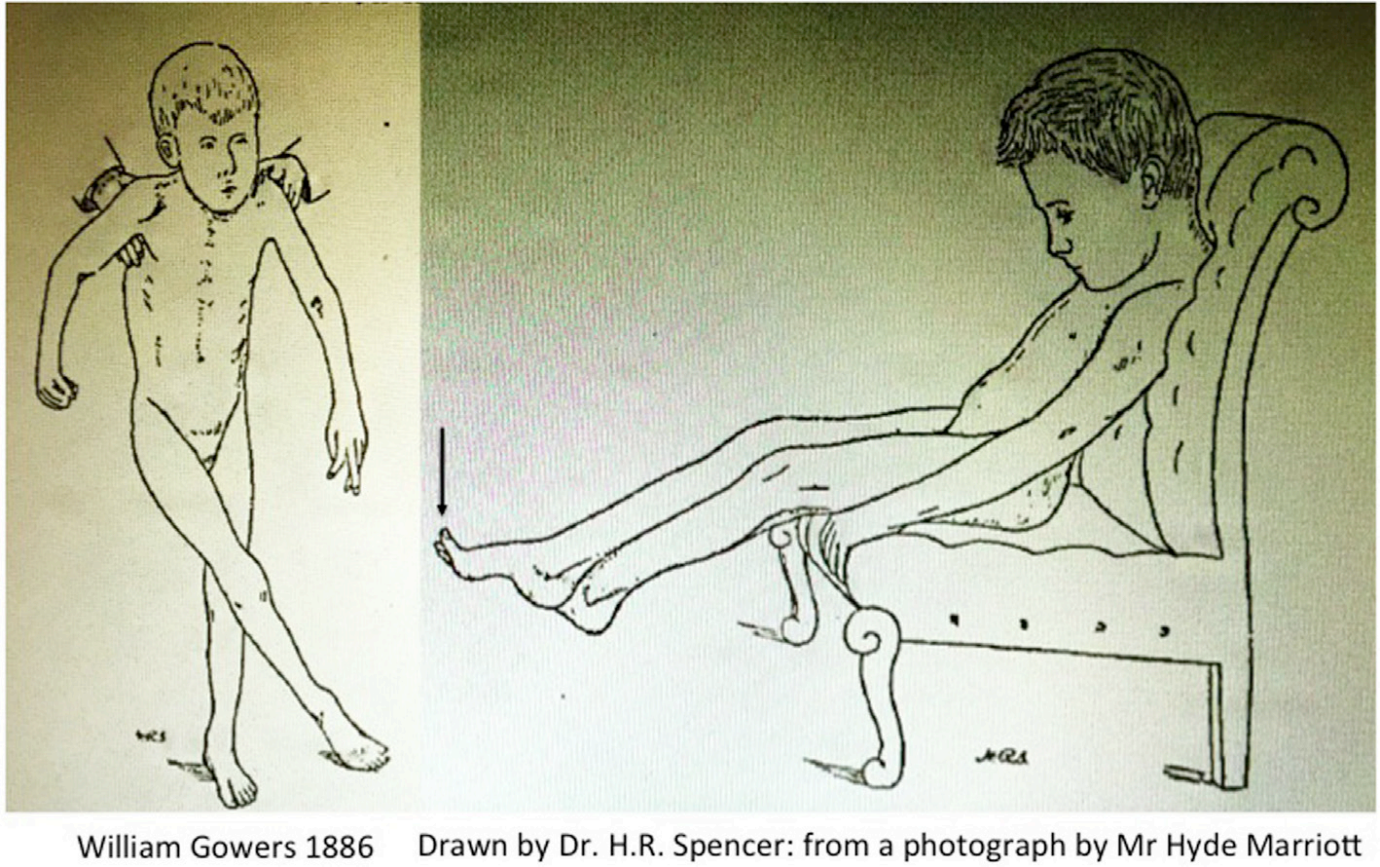
**Scissoring in cerebral palsy by Gowers (46)**.

Gowers produced one of the first clinical descriptions of a child with CP, which he attributed to pathology of the spinal cord arising during labour. This explains why this typical description of CP in *A Manual of Diseases of the Nervous System* is found in *Volume 1: Diseases of the Spinal Cord and Nerves* with the two iconic illustrations Fig. 117, “*child seated*” and Fig. 118 “*child in supported standing*” (46), reproduced here in Figure 2.

Despite the repeated attribution of the clinical syndrome to “spastic paraplegia,” Gowers’ full description leaves little doubt that the features also include unequivocal descriptions of dystonia and chorea:

The active contracture in the calf muscles, which most cases present, is a serious hindrance to walking even when the muscular power is sufficient. It is long before the attempt to walk overcomes the contracture. In most cases, however the child ultimately gains the power of walking, although much later than normal, and it often presents some peculiarity of gait, sometimes a tendency to “cross-legged progression” in which one foot gets over or in front of the other (Fig. 118), or a swinging oscillation of the body occurs which may persist to adult life. The infantile form may resemble very closely that which occurs in adults. There is the same extensor spasm and increase of all forms of reflex action. **As the child sits on the knee or a chair any sensory stimulus will make the legs shoot out in spasm (Fig. 117)**. But the spasm does not reach the extreme degree often attained in the common form. The excess of the knee-jerk is always distinct, **but a foot-clonus is not often to be obtained**, perhaps because the muscle-reflex mechanism related to the calf-muscles has not received the functional development that must result from the ever-recurring sequence of tension and contraction involved in walking.

The frequent observation of dystonic choreoathetosis is explicitly described in Gowers’ next sentences:

The arms do not present tonic spasm such as is seen in adult cases. There may be choreioid disorder of movement, spontaneous irregular movements with inco-ordination. In the cases that can be fairly called “spastic paraplegia,” the arm symptoms are slight. When considerable, the condition is usually termed “double athetosis” and its characters are described in diseases of the brain. [Gowers (46)]

The striking description of the “cross-legged progression” has been appropriated today as a hallmark of “spasticity,” a concept, which unjustifiably pervades clinical practice, and serves to characterize the dominant phenotype of CP. But this peculiar cross-legged posture is seldom subjected to an operational analysis.

Gowers clearly describes sudden jerking extension of the legs precipitated by non-specific afferent inputs (i.e., increased arousal); there is seldom if any elicitable clonus at the ankle; the feet are maintained in an equinovarus posture; the published Fig. 117 (Vol. 1, p. 333) clearly shows spontaneous extensor great toes, a feature notable enough for the sketch drawn by Mr. H. R. Spencer to include this feature when drawn from the photograph taken by Mr. Hyde Marriott; the cross-legged posture occurs chiefly when the child is held in supported standing, i.e., head upright and again there are spontaneous extensor great toes in Fig. 118 (Vol. 1, p. 334). Finally Gowers explains: “*There may be choreioid disorder of movement, spontaneous irregular movements with inco-ordination*.”

This leaves little doubt that Gowers was describing a coexisting movement disorder, not just paralysis with brisk reflexes, and the appearance of this description under the section “Primary Spastic Paraplegia” must be considered misplaced.

**Figure 3:**
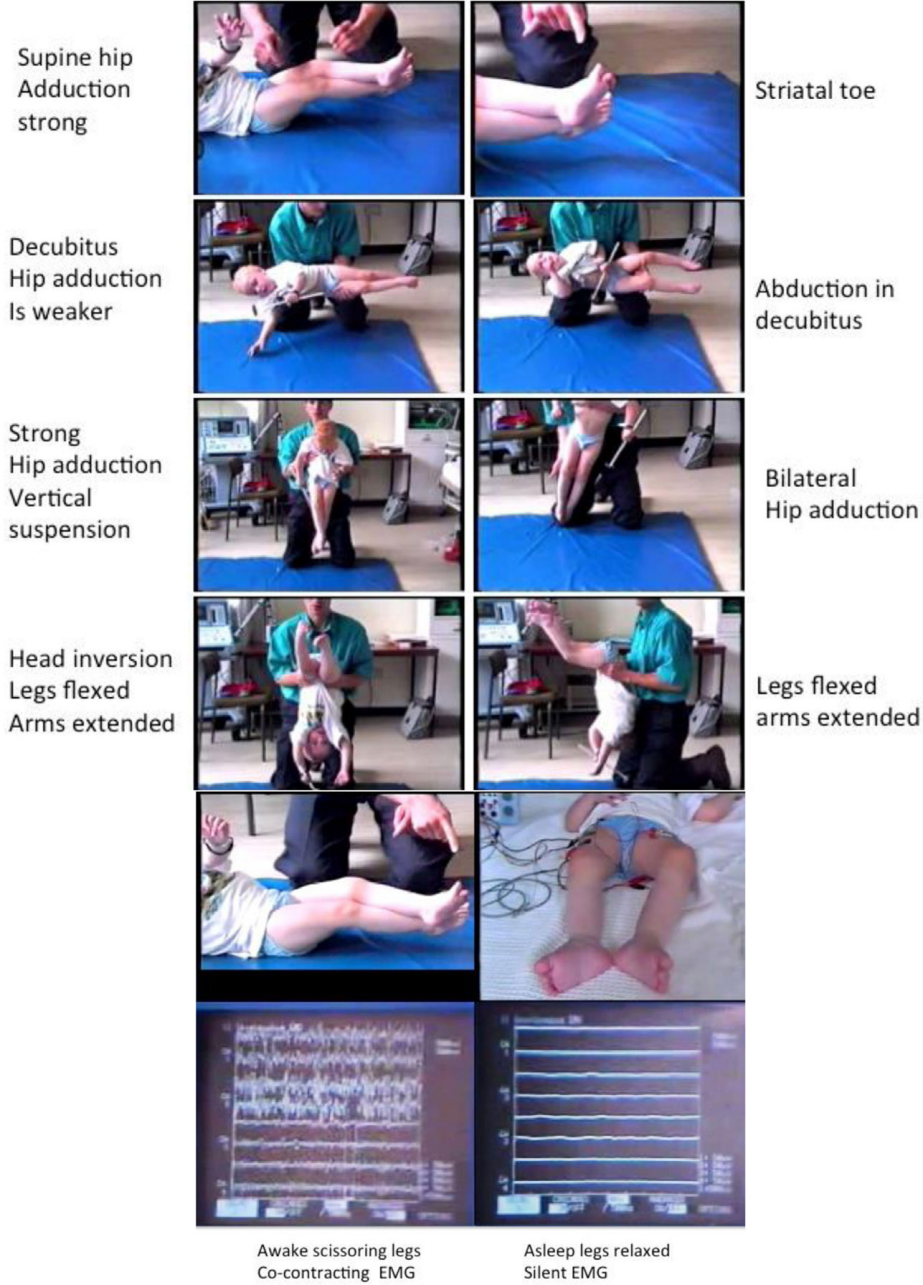
**Tonic labyrinthine responses (TLRs)**. Provoked by supine and vertical suspension. Abolished body inversion (held upside down) and by sleep. Three-year old ex-preterm child with shunted hydrocephalus. See figures for explanation. The TLR is what produces “scissoring” in many children and young people with CP. Although often considered a feature of the “spastic syndrome,” the TLR shares more in common with dystonia since it is associated with bilateral spontaneous extensor great toes also known as the “Striatal Toe” being a hallmark of dystonia. When the Babinski manoeuver is performed (stroking the sole of the foot firmly), the great toes flex. The TLR is also associated with cocontraction of agonist–antagonist muscles and this is abolished by sleep, another features of dystonia. *Operational definitions of the TLR*: Vertical suspension (top left) and supine lying (top right) bring out scissoring of the legs [adduction of the hips, extension of the knees, equinus foot posturing at the ankle, and spontaneous extensor (striatal) toes] (top panels). The legs cannot be passively separated (3rd panels from top) and move “*en bloc*.” Inverting the child produces abduction and flexion at the hips with flexion of the knees and reduced ankle equinus (4th panels from top). Sleep abolishes the scissoring of the TLR in the supine posture (bottom left) and the muscles are completely at rest as measured by surface electromyography (bottom right): the legs are completely relaxed and offer no resistance to passive movements. *Adapted from Dr. J.-P. Lin “Motor Assessment in Children with Cerebral Palsy*” *Ph.D. thesis, 1998, Edinburgh*.

This whole phenomenon has been reviewed under electromyographic recording (Figure 3) with specific attention to the following conditions:

Awake: in supine, decubitus, vertical suspension, and inverted suspension (Figure 3).
Awake and asleep in supine followed by waking up with EMG monitoring (Figure 3).

**Figure 4:**
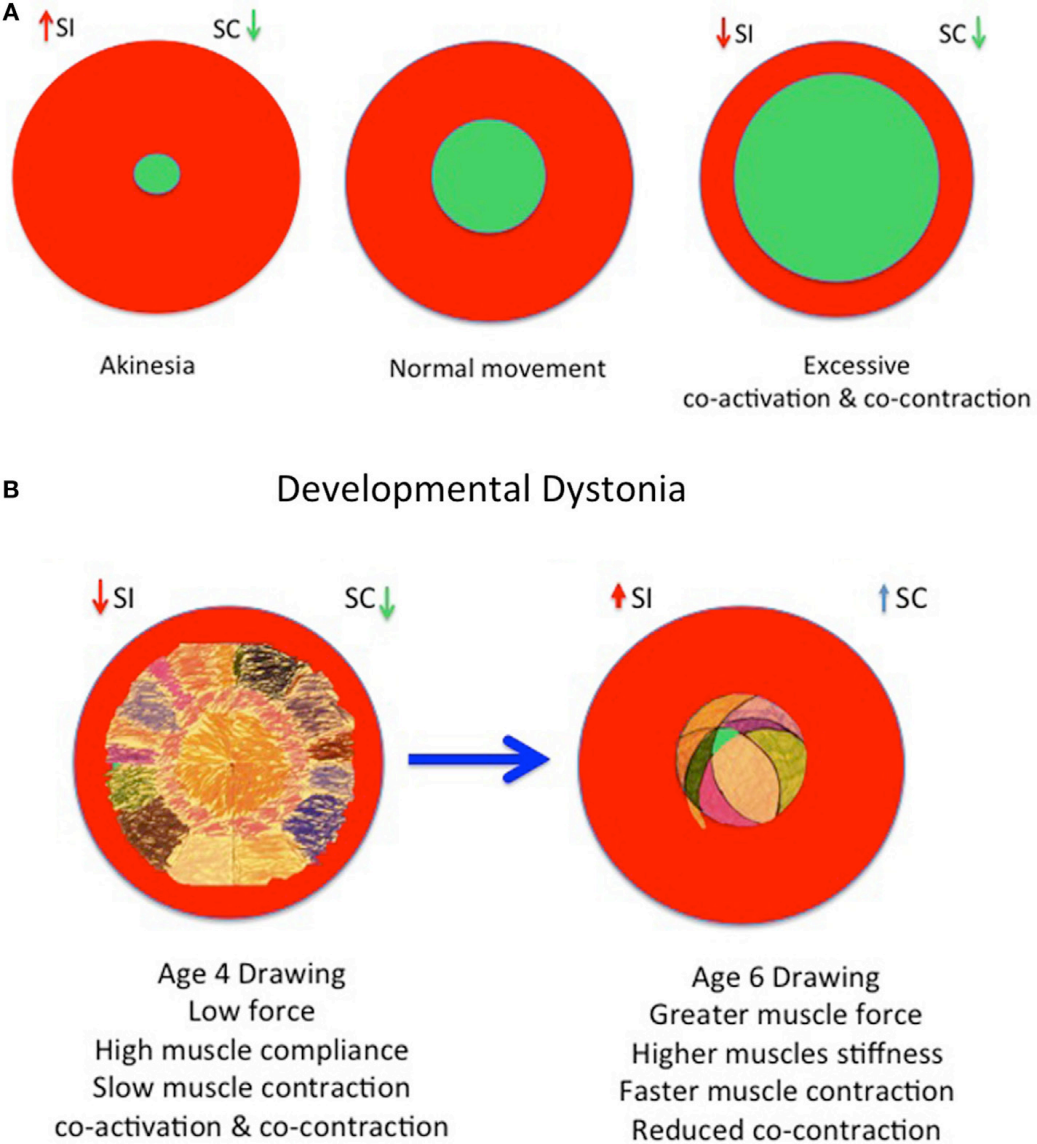

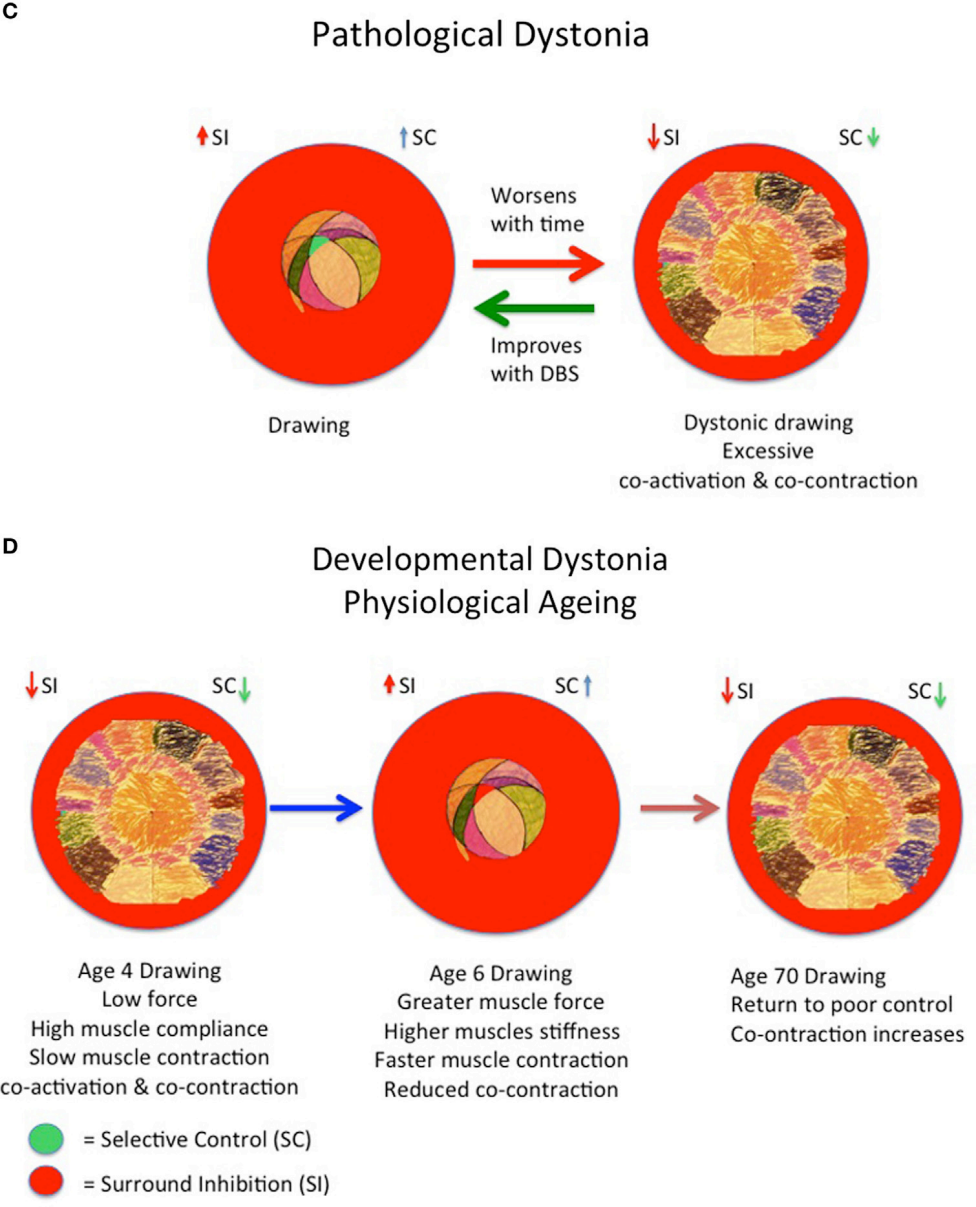
**Developmental model of physiological and pathological dystonia in young and elderly**. **(A)** Model of akinesia, kinesia, and hyperkinesia according to relative balance of selective control (SC) and surround inhibition (SI) with rise in cocontraction. **(B)** Increasing SC as increasing surround inhibition (SI) “prunes” excessive movements in childhood from age 3 to 6 years, respectively. **(C)** The onset of pathological dystonia is associated with reduced surround inhibition (SI) and reduced SC. **(D)** The elderly experience reduced SC and reduced surround inhibition (SI) with consequent loss of skilled performance. Adapted from Lin et al. (30), Mink (53), Tedroff et al. (35), and Graziaido et al. (36).

This careful observation demonstrates that the cross-legged posture of the legs is determined by arousal-wakefulness and exacerbated with increasing arousal-excitement. The cross-legged posture (now called “scissoring” of the legs) is completely inhibited by inverting the head and trunk, literally being held upside down, which also abolishes the tonic knee extension. This whole phenomenon being described by Denny-Brown (47, 48) and extensively reviewed by accessing the Denny-Brown collection of films (49).

Likewise the cross-legged posture, which is accompanied by spontaneous and continuous agonist–antagonist muscle contraction with the child in the awake, supine position (Figure 3) is also completely abolished by sleep, when the spontaneous EMG recordings cease altogether and the legs become completely flaccid at the hip and knee, ankle and externally rotated at the hip (Figure 3). Wakefulness-arousal immediately restores the scissoring leg posture (Figure 3).

None of the above-described phenomena are in any way related to the velocity-dependent increase in stretch reflexes described by Lance (50), the features being more consistent with a fixed dystonia (in contrast to rapid or phasic dystonia).

### Responses to Postural Perturbation and the Dystonic Response

Another significant developmental milestone appears to be the ability to adaptively respond to sudden perturbations of posture. This was elegantly studied in typically developing children and children with CP by Nashner et al. (51) using tilt platforms to apply sudden forward or backward tilts.

When tilted forwards or backwards, healthy children stabilize the ankle followed by the knee joint later: a “*distal-to-proximal*” muscle activation. In hemiplegic or diplegic CP “*proximal-to-distal*” activation sequences occur when tilted forwards or backwards, except in *uninvolved* legs of hemiplegic or ataxic children (51, 52) Nashner concluded:

The results of moving platform assessment support the contention that pathological changes in stretch reflex mechanisms associated with spasticity are secondary to the primary functional loss: namely, alterations in central and spinal programs which impose the appropriate temporal and spatial structures upon motor activities of the limb prior to the execution of the task (51).

Nashner is categorical: the response to perturbation in CP children is a maladaptive proximal to distal motor-activation ripple which acts first in muscles around the hip, momentarily leaving the knee and ankle relatively flail. This contrasts with the strategy of stabilizing ankle and knee joints before stabilizing the hip joint in healthy children. Inputs from the labyrinths (vestibular system) facilitate functional postures but are exaggerated in “*quadriplegia”* and “*diplegia”* according to head position.

Dystonia, rigidity, chorea, and hemiballismus reflect imbalances between desired and competing cerebello-thalamo-cortical-basal ganglia circuit activity causing a failure to inhibit unwanted movements through abnormal activation patterns of groups of striatal neurons (53, 54) within segregated and integrative connectivity patterns in the human basal ganglia (55). Posterior sensorimotor, middle cognitive–associative, and anterior limbic functions being respectively colocated in each basal ganglia structure (55). Understanding how we develop *habitual* as opposed to *goal-directed movements* (27) could explain why children with CP have difficulty producing successful habitual motor repertoires.

## A Need to Recognize That Many Different Patterns of Brain Injury Can Cause Dystonia: Emphasizing Dystonia as a Manifestation of a Disturbed Distributed Network

If we accept that dystonia emerges out of an initial developmental cocontraction pattern of movements and postures and that motor development arises from an *ab initio* hyperkinetic motor condition, it is not difficult to understand why so many disorders with seemingly completely different mechanisms result in dystonia.

Apart from the classical injuries of the developing brain (56), approximately 17–20% of children with CP have normal neuroimaging (57–59), which calls into question “*where exactly is the non-progressive injury to the developing brain*” said to be the defining hallmark of CP? A feature all the more intriguing when over 50% of children with “dyskinetic CP” have normal brain imaging (60).

This suggests that the brain in early dystonic motor disorders may fail to develop the efficient and functional connectivity networks underpinning normal motor development. By extension of this hypothesis, the child with early onset dystonia may be utilizing an under-pruned, over-arborized network of connections (rather than a damaged network). This calls into question therapeutic approaches centered on “curing” CP with stem cell therapies, for instance see Graham (44) for a recent discussion of the role of stem cells in CP. However, it is conceivable that stem cells, not only repair damaged areas of the brain but also facilitate activity-dependent plasticity, which could be associated with promoting functional connectivity.

Connectivity within the brain of the child with dystonia, may be measured by whole white matter fractional anisotropy (FA) estimations using diffusion-weighted imaging methods with the technique of MRI Diffusion Tensor Imaging. This provides information about white matter microstructure integrity and is derived from the differential diffusion of water molecules along fiber tracts. The FA value from 0.0 to 1.0 represents the likelihood of water diffusing along fibers (FA maximum = 1.0) or water diffusing randomly in all directions because there are no fibers (FA minimum = 0.0). The FA value may therefore be a useful marker of the connectivity of the brain: higher FA values corresponding to greater structural connectivity and integrity of the white matter fiber tracts within the brain and *vice versa*. For instance, in a cohort of children selected on clinical, conventional radiological, and neurophysiological assessments (61, 62) preparatory to management of dystonia with DBS or ITB, the mean DBS-group FA value was >0.5, but the FA value was <0.5 in the ITB group (63). Such statistical imaging parameters may in the future, with neurophysiological parameters become a means of appropriately stratifying children with dystonia into prognostic groups suitable for intervention with DBS or ITB.

## The Need to Recognize Non-Motor Symptoms in Childhood Dystonia

The focus on the classic motor symptoms of dystonia, i.e., involuntary muscle contractions and abnormal postures has left a gap in research on the non-motor aspects of the dystonic disorders such as abnormalities in sensory and perceptual functions, neuropsychiatric disturbances, and sleep. The comorbidity of non-motor symptoms is increasingly recognized in adults with dystonia (64, 65). It is not known, if these non-motor symptoms, including poor sleep patterns (41), are intrinsic to dystonia comorbidities or secondary to the dystonia disorders themselves. Understanding the role of non-motor symptoms in childhood dystonia has potential implications for clinical assessment tools; pre- and post-surgical interventions, e.g., deep brain stimulation (DBS), and treatment targets. Moreover, these non-motor symptoms play a vital role in determining quality of life. Although research has begun to investigate these non-motor phenomena in typically developing children and autistic children, no systematic evaluation of non-motor symptoms exist for the childhood dystonia population.

A recent comparison of the relationship between Burke Fahn Marsden Dystonia Rating Scale-motor (BFMDRS-m: measuring dystonia severity) and three functional measures, the Gross Motor Function Classification System (GMFCS), the Manual Abilities Classification System (MACS) and Communication Function Classification System (CFCS) showed an extremely good linear correlation between the BFMDRS-m and the GMFCS, indicating an approximately 20–25 absolute BFMDRS points between each of the five divisions of the GMFCS (Grade I = athletic, Grade V = no mobility at all). However, although the BFMDRS-m correlated well with the MACS, the BFMDRS did not discriminate well between Grades I, II, and III of the five MACS grades, suggesting that non-dystonic variables also impair manual abilities: e.g., processing skills or lack of opportunity to practice skills (66).

Further work on the non-motor aspects of living with dystonia is urgently required. These non-motor aspects of dystonia could relate to abnormal sensory processing, body image, cognitive issues, and the social context of physical dependency and lack of opportunity for independence.

Additional areas for exploration in dystonia include the role of mirror neurons and the ability or otherwise to learn new motor skills through observed movements when living and growing as a child with dystonia.

## Measuring What Matters Most to Children with Dystonia is also an Unmet Need

Most studies fail to report what matters most to children with dystonia and their carers: reduction in pain, improvements in activities of daily living, and manual function (67, 68). This is a particularly important failing since dystonia worsens in approximately 2/3 of cases and remains severe in a further 1/3 of cases referred for further management after conventional medication (1).

## The Unmet Need of Preventing Deformity in Childhood Dystonia

Fixed musculoskeletal deformity (FMD) appears inexorably with each successive 5 years of living with dystonia in children but is most marked in the secondary dystonias (69). For instance the median age of FMD in primary dystonia is >21 years (95% CI: 16–21 years) but falls to 6 years (95% CI: 5.0–6.1 years) in secondary dystonias, and 7.4 years (95% CI: 4.4–10.4 years) in heredodegenerative dystonias.

Urgent means are required to forestall the onset of deformity in childhood secondary dystonia, particularly in CP children with a GMFCS level V, since secondary dystonias are very vulnerable to early fixed deformity, certainly before the age of five and well before the end of primary school education (69).

## Exploration of Better Pharmacological Approaches to Managing Dystonia in Childhood as an Unmet Need

Although several medications are regularly used in the first-line management of dystonia, the evidence to support pharmacological agents in childhood dystonia is weak (70). Trihexyphenidyl has some evidence of efficacy to support its use in adults with dystonia. In a retrospective cohort of 278 children with dystonia referred to a single center from all over the United Kingdom and Republic of Ireland, medication use had been prospectively gathered including adverse drug reactions (ADR) (71).

The commonest drugs used were baclofen (118/278: 42.4%), trihexyphenidyl (98/278: 35.2%), l-dopa (57/278: 20.5%), and diazepam (53/278: 19%). Choice of medication appeared to be influenced by dystonia etiology (71). ADR had occurred in 171/278 (61.5%) of children: the commonest drugs responsible for ADR being trihexyphenidyl (90/171: 52.3%), baclofen (43/171: 25.1%), and l-dopa (26/171: 15.2%) (70). Unfortunately, pharmacological management of dystonia in children is often disappointing or not tolerated (70, 71). No medication is licensed for the management of dystonia in childhood (70). This includes new conceptual approaches to mechanisms of relieving dystonia. Gabapentin has been reported to be beneficial in relieving dystonia in a relatively large open trial in children with often severe dystonia, which was liable to disrupt night sleep, impair the capacity to tolerate sitting comfortably, reduce activities of daily living, disrupt mood and behavior, and very often associated with severe pain (72). Outcomes were measured using the Dystonia Severity Assessment Plan (DSAP) (42) and the activities of daily living were mapped on a scale of 0–4 using the WHO International Classification of Function where 0 = no difficulty and 4 = maximum difficulty most of the time (72). Gabapentin may thus become a new, “*re-purposed*” medication for routine management of dystonias in childhood subject to further studies with a view to obtaining a license for gabapentin liquid and tablets/capsules in childhood dystonia.

## The Need to Adequately Manage Life-Threatening Dystonias in Childhood Including “Brittle Dystonia” and SD

In a recent study of over 50 cases of SD in children and young people, SD was reported to be life-threatening in up to 10% of cases (45) from multi-organ failure but also because the use of muscle relaxants such a benzodiazepine infusions and high-dose baclofen can cause respiratory depression.

The use of a scale such as the DSAP (42, 43, 72) may help measure change and recovery together with drug and therapeutic efficacy, by promoting sleep as a major strategy for switching off dystonia rather than resorting to muscle relaxants and ventilator support. The importance of the sleep chart must be emphasized in monitoring the well being of those children with dystonia who are liable to experience frequent episodes of severe dystonia (brittle dystonia) requiring hospital admission often in high-dependency units or pediatric intensive care units.

Early features of decompensating dystonia may be detected using the DSAP. DSAP Grade I = sitting comfortably all day; Grade II = unable to tolerate sitting; Grade III = unable to sleep at night; Grade IV = early metabolic decompensation, sweating, and mild rhabdomyolysis; and Grade V = full SD with multi-organ failure. The change in DSAP can prompt seeking advice and intervention before dystonia decompensation, as well as a means of monitoring progress toward recovery. DBS now has an important role in the management of SD (43, 45).

## There is an Unmet Need to Develop Good Biomarkers for Dystonia in Childhood

We have already looked at non-invasive neurophysiological variables throughout this review, most of which will depend on clear operational definitions for measurement and interpretation of clinical significance. Such measures lead to categorical and quantitative data, e.g., the latency of central motor conduction times (61, 62). White matter integrity as measured by the FA value (62, 63) may be used to partition cases most likely to benefit from DBS or ITB (63) and thereby improve the prognosis for a favorable outcome from DBS for dystonia along with somatosensory-evoked potentials (SSEP) (J-P Lin unpublished results).

Other imaging modalities, such as 18-fluorodeoxy Glucose Positron Emission Tomography (FDG-PET-CT) imaging, may also permit individual and group analyses of cerebral function in dystonia using statistical parametric mapping techniques which may shed light on how dystonia affects brain function and highlight differences between different causes of dystonia such as between primary dystonia and dystonia arising from neurodegeneration with brain iron accumulation (NBIA) caused by the pantothenate kinase-2 (PANK-2) mutation (73). Other dystonias may reveal other resting state glucose metabolism patterns reflecting functional connectivity patterns in children with and without conspicuous brain injuries (Lin, unpublished observations).

More invasive studies, such as globus pallidus internus microelectrode recordings (GPIMER) have revealed differences in the proportion of irregular and bursting cells in children undergoing DBS for dystonia (74). Children suffering from NBIA, mostly secondary to PANK-2 disease had mostly regular GPI neuronal firing patterns and the highest mean GPIMER neuronal instantaneous firing frequencies, with no bursting cells. In contrast, the secondary dystonias had the slowest mean and median GPIMER instantaneous firing frequencies, but the same relative proportion of bursting cells as primary dystonias (74). In addition, the GPIMER for postneonatal secondary dystonias had higher instantaneous firing frequencies than found in the GPIMER firing frequencies in children with perinatal causes of dystonia. Such GPIMER firing frequencies correlated positively with follow-up changes in the BFMDRS at 1 year after DBS surgery (74).

## An Unmet Need for Rapid Diagnostic Testing in Childhood Dystonias and Early Recognition of Childhood-Onset Dystonia

Next generation DNA sequencing should reduce the wait for molecular confirmation of a genetically inherited movement disorder, including the dystonias. Currently, the clinical reality is that there are huge time-lapses between the clinical suspicion of a genetic basis to dystonia and genetic confirmation. This lack of clarity delays clinical decision-making and sustains the anxiety surrounding every “unsolved” case of dystonia. The possible overlap between the genetic dystonias and a possible perinatal mechanism is especially likely when brain imaging is unremarkable.

Another diagnostic dilemma is the advent of multiple clinical phenotypes arising from a single genetic disorder, a phenomenon known as phenotypic pleiotropy, which may present, for instance, as seizures in infancy and a movement disorder many years later.

Genetic heterogeneity and phenotypic pleiotropy have further supported the case for a basic neurogenetic screen in children with neurological disorders (75), even in those children in whom there appear to be brain scan imaging changes and particularly in those CP cases with apparently “normal” brain scans.

Such a widespread screen would identify more genetic cases and broaden our phenotypic understanding of gene functioning and aid population modeling of dystonic disorders in childhood.

Dystonia in children often requires time to be recognized, assessed, and eventually diagnosed, perhaps not surprisingly, given the immense neurodevelopmental backdrop of cocontraction underpinning early human motor patterns.

With regard to clinical presentation, the typical site of onset of childhood isolated dystonia is in the lower limbs; a considerable proportion of cases subsequently generalize to involve the trunk and upper limbs, while onset in the cervical, oromandibular, and laryngeal regions are less frequently encountered and should always lead to ruling out secondary causes of dystonia, such as NBIA, cerebral neoplasia, or disorders of the cranio-cervical junction.

Abnormal postures of the ankle, foot or both, or an abnormal gait pattern due to mobile lower limb dystonia can take a long time to be recognized as a primarily neurological issue. In this regard, there is an unmet need to bridge the gaps between orthopedics and neurology, thus widening the knowledge and awareness by other professionals of childhood dystonia. This may hopefully allow children to be promptly assessed by child neurologists and referred to dedicated movement disorders clinics.

Early clinical diagnosis not only leads to early management but also to early counseling to patients’ families. Children affected by dystonia are mostly sporadic cases, namely no additional family members are affected and their parents are healthy. A recurrent question made by children’s families in pediatric movement disorders clinics is about the probability of other children being affected by the same disorder. This question still remains unanswered in many cases; in fact, it is impossible to estimate the risk of transmission of these dystonias to offspring without a definite genetic diagnosis in the proband. Recent advances in genetic techniques have allowed the recognition of an increasing number of genes underlying dystonia, but most of the genes discovered in the past 4 years seem to be almost exclusively found in adult-onset cases (e.g., GNAL, ANO-3) and are apparently a very rare cause of dystonia. For example, only 53 patients with GNAL mutations have been reported since the discovery of this gene in 2012 (76), and only 10 ANO-3 positive patients have been fully characterized clinically (77). Moreover, no dedicated studies aimed at assessing the frequency of mutations in these genes have been performed in pediatric cohorts of patients. Thus, there is a current unmet need to better characterize the genetic causes of dystonia in children by analyzing not only well known genes such as DYT-1 and DYT-5 but also recently discovered genes that have primarily been characterized in adult patients, or which exhibit phenotypic pleiotropy on the one hand (75), and to account for the growing recognition of genetic heterogeneity in the causes of dystonia on the other (75). This is ideally possible by using next generation sequencing platforms containing all the dystonia-related genes reported so far, but even by using this powerful tool, a significant proportion of pediatric patients still remain genetically undiagnosed. This reinforces the hypothesis that dystonia is a complex and genetically heterogeneous disorder and additional efforts are needed to share patients’ DNA samples among research centers to discover new dystonia-causing genes. The availability of genetic tests in clinical practice will be crucial to widen our knowledge of dystonia, clinical phenotypes, and disease course in affected patients. However, the rising number of new gene discoveries and the movement disorders they may cause provide a formidable task for even specialist clinicians to keep up with (78), even though genetic experts are doing their best to provide rational systems of movement disorder gene classifications (79).

## Developing Coherent, Networked Research Strategies for Diagnosing and Stratified Management of Dystonias in Childhood: An Unmet Need

It is not enough to focus on very specific, mostly rare, and monogenic dystonias. Another approach, as described in this text, is to look at the shared characteristics of all childhood dystonias to find techniques that can ameliorate the lives of children, especially with early-onset dystonias. This approach requires a more thorough analysis of the stages of motor development, including key elements underpinning human motor development and function susceptible to *disruption, suppression* or *arrest* by uncontrolled early dystonia.

Monogenic isolated dystonias, dystonia plus, and even progressive dystonias often have a period of normal early motor development which offers the possibility that functional skills may return more easily and quickly if dystonia can be reduced, for instance with neuromodulation by DBS. Conversely, this is less likely to occur in the absence of an early period of normal motor development before dystonia-onset, as is typical of isolated or mixed secondary dystonias such as dystonic CP (1). This leads us to consider a paradox: dystonia may be considered to be a product of excessive neuronal plasticity and inadequate surround inhibition (38, 39). But cases that become DBS-dependent, often requiring high DBS currents to maintain dystonia suppression, are said to possess too little cerebral plasticity to produce long-lasting “DBS-off” dystonia suppression (80, 81). We thus need to understand how cerebral plasticity; long-term potentiation and long-term depression can be harnessed or focused in all these different childhood dystonias.

An additional factor is the burden of growing and developing with continuously disruptive dystonic movements and postures leading to a concern that the “proportion of life lived with dystonia” (dystonia duration/age) can adversely compromise future motor skill acquisition and reduce the neuromodulation efficacy of interventions such as DBS through lost opportunities for activity and participation (1) since living with dystonia for a long time may reduce the likelihood of reducing dystonia (82).

Dystonia in childhood remains largely a physiological mystery, all the more complex because it occurs in the context of a developing brain. A number of theoretical approaches have been advanced to understand how dystonia and other early-expressed neurological disorders can be understood. These include the neuronal group selection theory proposed by Hadders-Algra (83), which attempts to model how groups of neurons within a network establish patterns of behavior. But neurodevelopmental therapy, in the form of Sensory Integration, the dominantly promoted management for motor disorders in children since the 1970s, has not proven beneficial when closely evaluated (84).

A recent review of a framework for understanding many different early onset neurodevelopmental disorders examines the “critical” and “sensitive” windows of brain plasticity that depend on expected inputs during “critical periods” which may be disrupted by other inputs during later “sensitive periods” when programed developmental outcomes are thwarted by neurological disorders (85). For instance, the visual cortex expects visual inputs in a critical period, which if missed, leads to visual agnosia, e.g., ocular defects may produce amblyopia in uncorrected squints. However, auditory and tactile stimuli may have greater cortical representation and help compensate for the blindness. Similarly, the auditory cortex requires early auditory inputs to develop language. Congenital deafness must be corrected early, e.g., within 5 years of life but typically as early as possible if the child is to develop language skills (86, 87).

Developmental dystonias and epilepsies take their toll on the developing brain by subverting this delicate plastic organization, which require timely interventions that can restore functional adaptive plasticity.

Collaborative networks are required using common definitions and meaningful outcome measures to develop adequate dystonia stratification for therapeutic choice and individual patient prognosis (88, 89) within a framework of the International Classification of Function (90) with measures that are meaningful to children with dystonia and their families (66–68). New work is differentiating the relative impact of dystonia on function compared to choreoathetosis and this reveals that dystonia is the most important factor associated with neurodisability (91). These studies must be augmented with new, clinically relevant studies to help parents and carers, children, and young people make appropriate decisions in relation to complex neurosurgical interventions such as DBS (where appropriate) (92–95). But for any field to prosper we must be prepared to look elsewhere for examples of success and also applications of successful neuromodulation interventions, for instance, in the management of chronic pain (96) and bladder and bowel incontinence (97) where neuromodulation can make a significant positive impact. We must also evaluate the childhood brain and define stimulation targets in appropriate targets of the motor conectome in children (98) and ensure preservation of cognitive (99) function and accept the technical challenges of new imaging techniques to safely work with neuromodulation systems (100).

In implementing new technologies we must take care to integrate these within a framework of close cooperation with children, families, carers, and colleagues, using “the broad clinical gaze” which avoids a merely technocratic solution by working in the best interests of the child (101). A major approach advocated in this manifesto, is to study what all dystonias share in common since this must represent what is biologically fundamental to the dystonic state. Such an approach may produce surprising and novel ways of dealing with the current “unmet needs in dystonia.” This “manifesto” should stimulate and facilitate clinical networking, appropriate diagnostic “stratification” and functional assessments for precision medical and functional neurosurgical intervention selection and research in childhood dystonias.

## Ethics Statement

Photographs of clinical demonstrations relating to physiology of the TLR were performed by J-PL as part of his Ph.D. thesis “Motor Assessments in Cerebral Palsy” at the Royal Hospital For Sick Children, Edinburgh and were granted approval by the Lothian and Borders Health Board Research and Ethics committee. Pediatric/Reproductive Medicine Ethic of Medical Research sub-committee: Protocol 10/92: “The objective assessment of muscle tone, posture and movement in cerebral palsy” 16.09.92 (ref CJA/JHNL). Other clinical photographs are published with clinical consent within Guy’s and St Thomas’ NHDS Foundation Trust.

## Author Contributions

J-PL conceived the project, wrote the first draft, added some clinical examples as demonstrations, and critically reviewed the manuscript. NN critically reviewed the manuscript.

## Conflict of Interest Statement

The authors declare that the research was conducted in the absence of any commercial or financial relationships that could be construed as a potential conflict of interest.

## Appendix

### Panel 1. A Manifesto of Unmet Needs in Isolated Childhood Dystonias

The following headings represent some of the unmet needs in understanding and managing childhood dystonia:

1. There is a need to recognize the importance of early experience of movement for the fetus and infant and environmental effects on this early development.
2. A need to recognize the link between childhood dystonias and patterns of movement and postures in the infant and toddler. This involves recognizing the key patterns of emergent motor development and applying operational definitions that enable recognition of these key patterns, including understanding the link between developmental dystonia and pathological dystonia at all ages of life.
3. An unmet need to recognize the similarities and differences between developmental cocontraction, task-dependent cocontraction, the pathological cocontraction of dystonia, observations of selective motor control, and surround inhibition in children, the elderly, and in dystonic individuals.
4. An unmet need to recognize that dystonia is abolished by sleep in children and adults and may therefore be used as a diagnostic criterion for dystonia as well as therapeutically, for instance in status dystonicus.
5. The need to recognize dystonia which pervades cerebral palsy and reverse the over-investment in the “spastic model” of cerebral palsy in medical teaching and training on motor disorders in childhood arising from historical roots at a time when dystonia was not commonly recognized leading to an under-recognition of the true prevalence of dystonic cerebral palsy including a reexamination of the nature of the TLR.
6. A need to recognize that many different patterns of brain injury can cause dystonia: emphasizing dystonia as a manifestation of a disturbed distributed network.
7. The need to recognize non-motor symptoms in childhood dystonia.
8. Measuring what matters most to children with dystonia is also an unmet need.
9. The unmet need of preventing deformity in childhood dystonia.
10. Exploration of better pharmacological approaches to managing dystonia in childhood as an unmet need.
11. The need to adequately manage life-threatening dystonias in childhood including “brittle dystonia” and status dystonicus.
12. There is an unmet need to develop good biomarkers for dystonia in childhood.
13. An unmet need for rapid diagnostic testing in childhood dystonias is the need for an early recognition of childhood-onset dystonia.
14. Developing coherent, networked research strategies for diagnosing, and stratified management of dystonias in childhood: is an unmet need.

### Panel 2. Dystonia, Dyskinesia, and Hypertonus

Dystonia is defined as a movement disorder characterized by sustained or intermittent muscle contractions causing abnormal, often repetitive, movements, postures, or both. Dystonic movements are typically patterned and twisting, and may be tremulous. Dystonia is often initiated or worsened by voluntary action and associated with overflow muscle activation. Dystonia is classified along two axes: clinical characteristics, including age at onset, body distribution, temporal pattern and associated features (additional movement disorders or neurological features), and etiology, which includes nervous system pathology and inheritance. The clinical characteristics fall into several specific dystonia syndromes that help to guide diagnosis and treatment. Albanese et al. (102)

The terms Dyskinesia and Hypertonus occur throughout the literature on CP and have overlapping but also distinct meanings

### Dyskinesia

“*Dyskinesia*” or “dyskinetic” is a term originating from the Surveillance of Cerebral Palsy in Europe collaboration and has been used in many studies on CP which encompasses non-spastic and non-ataxic features including cases of *dystonia* or choreoathetosis in isolation or both in varying proportions. The term “*dyskinetic cerebral* palsy” implies CP dominated by *dystonia, choreoathetosis*, or both (103).

### Hypertonus

The *Task Force on Childhood Motor Disorders*^104^ is a North American consortium which uses the term “*hypertonus*” as a collective term for disorders of tone which requires further sub-division into “*spasticity*,” “*dystonia*” and “*rigidity*”:

“Spasticity” is defined as hypertonia in which 1 or both of the following signs are present:

(1) resistance to externally imposed movement increases with increasing speed of stretch and varies with the direction of joint movement, and/or
(2) resistance to externally imposed movement rises rapidly above a threshold speed or joint angle.

“Dystonia” is defined as a movement disorder in which involuntary sustained or intermittent muscle contractions cause twisting and repetitive movements, abnormal postures, or both.

“Rigidity” is defined as hypertonia in which all of the following are true (104):

(1) The resistance to externally imposed joint movement is present at very low speeds of movement, does not depend on imposed speed and does not exhibit a speed or angle threshold;
(2) Simultaneous cocontraction of agonists and antagonists may occur, and this is reflected in an immediate resistance to a reversal of the direction of movement about a joint;
(3) the limb does not tend to return toward a particular fixed posture or extreme joint angle; and
(4) voluntary activity in distant muscle groups does not lead to involuntary movements about the rigid joints, although rigidity may worsen.

### Panel 3. Causes of Cocontraction in Children

(a) Developmental cocontraction.
  Fetal and early postnatal postures.
  High muscle–tendon compliance.
  Slow muscle contraction and relaxation characteristics.
  Weak force generation.
  Reciprocal excitation due to co-innervation of agonist–antagonist pairs.
  “wrap-around” muscle activation in gait cycle of infants and young children.
  Joint-synchronies: e.g., triple-flexion hip, knee, and ankle joints till age 2 years.
  Developmental crouch posture of infant and toddler.
  Fog posturing performing unfamiliar tasks: all 4-year olds and 25% 16-year olds when asked to walk on toes, heels, lateral border, and in-step of feet.
    Corticomuscular coherence amplitude modultion^1^:
    Reduced in the children and elderly and linked to greater cortical recruitment for everyday tasks.
(b) Task-dependent cocontraction.
  Increasing walking speed leads to cocontraction.
    Children with bilateral CP tend to hurry when walking thus exhibiting more task-dependent cocontraction.
(c) Pathological cocontraction.
  Dystonia:
    Associated with “reduced surround inhibition” and “increased plasticity” (see text footnote 1).
    Dystonia is abolished by sleep (see Figure 3).
    Pathological crouch posture.
    Involuntary synergies during voluntary tasks (see text footnote 1):
      Increased mean β-ERD.
      Reduced γ-event-related synchronization.
(d) Tonic labyrinthine postures.
  Alterations of position of head in space produces cocontraction which is abolished by sleep (see Figure 3).
